# Knowledge and practices about multidrug-resistant tuberculosis amongst healthcare workers in Maseru

**DOI:** 10.4102/phcfm.v7i1.774

**Published:** 2015-03-27

**Authors:** Ntambwe Malangu, Omotayo D. Adebanjo

**Affiliations:** 1 Department of Epidemiology & Biostatistics, University of Limpopo, Medunsa Campus, South Africa

## Abstract

**Background:**

To date, no study has been found that described the knowledge and practices of healthcare workers surrounding multidrug-resistant tuberculosis (MDR-TB) in Lesotho.

**Aim and setting:**

This study was conducted to fill this gap by investigating the knowledge level and practices surrounding MDR-TB amongst healthcare workers at Botsabelo Hospital in Maseru, Lesotho.

**Method:**

This was a cross-sectional survey conducted by means of a questionnaire designed specifically for this study. Data collected included sociodemographic and professional details; and responses to questions about knowledge and practices regarding MDR-TB. The questions ranged from the definition of MDR-TB to its treatment. Respondents’ practices such as the use of masks, guidelines and patient education were also assessed.

**Results:**

A response rate of 84.6% (110 out of 130) was achieved. The majority of participants were women (60%), married (71.8%) and nursing staff (74.5%). Overall, less than half (47.3%) of the participants had a good level of knowledge about MDR-TB. With regard to practice, about 83% of participants stated that they used protective masks whilst attending to MDR-TB patients. About two-thirds (66.4%) reported being personally involved in educating patients about MDR-TB; whilst about 55% stated that they referred to these guidelines.

**Conclusion:**

The level of knowledge about MDR-TB amongst healthcare workers at the study site was not at an acceptable level. Unsafe practices, such as not wearing protective masks and not referring to the MDR-TB treatment guidelines, were found to be associated with an insufficient level of knowledge about MDR-TB. An educational intervention is recommended for all healthcare providers at this facility.

## Abstrait

**Connaissances et pratiques du personnel de santé de Maseru au sujet de la tuberculose multi-résistante**.

**Contexte:**

A ce jour, on n'a pas trouvé d’étude qui décrive les connaissances et pratiques des professionnels de la santé sur la tuberculose multi-résistante (MDR-TB) au Lesotho.

**Objectif et cadre:**

Cette étude a été menée pour combler cette lacune en étudiant le niveau des connaissances et les pratiques relatives à la MDR-TB parmi le personnel de santé à l'Hôpital Botsabelo de Maseru, Lesotho.

**Méthode:**

Cette étude transversale a été effectuée au moyen d'un questionnaire conçu spécifiquement pour cette étude. Les données collectées comprennent des informations sociodémographiques et professionnelles; et des réponses à des questions sur les connaissances et pratiques relatives à la MDR-TB. Les questions allaient de la définition de la MDR-TB à son traitement. Les pratiques des personnes interrogées telles que l'utilisation de masques, les directives et l’éducation des patients ont aussi été évaluées.

**Résultats:**

On a obtenu un taux de réponse de 84.6% (110 sur 130). La majorité des participants étaient des femmes (60%), mariées (71.8%) et des infirmières (74.5%). En tout, moins de la moitié (47.3%) des participants avait un bon niveau de connaissances sur la MDR-TB. En ce qui concerne la pratique, environ 83% des participants ont déclaré qu'ils utilisaient des masques protecteurs pendant qu'ils s'occupaient des patients souffrant de MDR-TB. Environ les deux tiers (66.4%) ont déclaré qu'ils s'occupaient personnellement d’éduquer les patients sur la MDR-TB; alors que 55% ont dit qu'ils se référaient à ces directives.

**Conclusion:**

Le niveau des connaissances sur la MDR-TB parmi le personnel de santé sur le lieu de l’étude n’était pas à un niveau acceptable. Des pratiques dangereuses, comme de ne pas porter de masque protecteur et de ne pas se référer aux directives de traitement pour la MDR-TB, étaient associées à un niveau de connaissances insuffisant sur la MDR-TB. Il est donc recommandé d'organiser une formation éducative pour tous les professionnels de santé de cet établissement.

## Introduction

Lesotho is one of the countries with the highest *per capita* incidence of tuberculosis (TB) in the world. With 637 incident cases of TB per 100 000 people, it was the fifth most affected country by 2006.^1^ Despite having achieved a high detection rate of 84% and adopted the directly observed therapy short-course (DOTS) strategy, several cases of multidrug-resistant tuberculosis (MDR-TB) had been reported since 2005. By 2009, a least 259 cases of MDR-TB had been identified in a total population of about 1.8 million people.^2,3^ Given this alarming figure, it is imperative that healthcare providers in Lesotho be knowledgeable about MDR-TB and be able to adopt practices to curb further transmission of this serious condition.

Although it is generally assumed that healthcare service workers (HCWs) know about MDR-TB and its implications, the evidence from several studies worldwide have found that HCWs do not always have sufficient knowledge or the correct positive attitude; and do not exhibit acceptable practices regarding prevention and treatment of MDR-TB.^4,5,6,7,8^ To date, no study has been found describing the knowledge and practices of HCWs with regard to MDR-TB at Botsabelo Hospital in Maseru, Lesotho.

This study was conducted to fill this gap by investigating the knowledge level and practices surrounding MDR-TB amongst HCWs at this health facility. It is hoped that the findings from the study could be used by decision makers and institutional managers so as to design and implement interventions to address the shortcomings that were identified.

## Research methods and design

### Study design

This cross-sectional study was conducted by means of a questionnaire designed for the study, taking into account other published questionnaires and the World Health Organization's MDR-TB management guidelines.^9^ Besides sociodemographic and professional data, knowledge about MDR-TB was assessed by asking questions about the definition of MDR-TB, its aetiology, diagnosis, symptoms and treatment modalities. The knowledge level was categorised as good if a respondent scored at least 80%; and insufficient if the score was less than 80%. Participants’ practices with regard to MDR-TB were assessed by asking them whether they possessed a copy of the MDR-TB treatment guidelines, whether they referred to these guidelines, whether they were involved in educating patients on MDR-TB and whether they reported using protective masks whilst attending to patients with MDR-TB.

### Study population

This questionnaire was semi-structured, anonymous and self-administered. Given the small number of the targeted population, the questionnaire was sent to all 130 HCWs working at Botsabelo hospital, based on the information from the hospital human resources database. This referral hospital in Maseru district has 24 beds for MDR-TB patients and serves nine other districts.

Specially-designed collection boxes were placed in convenient places for participants to drop off the completed questionnaires. The boxes were in place from 23 September to 15 October 2010.

### Data analysis

The field worker collected the returned questionnaires from the boxes, checked each one of them for completeness and numbered them. Data were captured into a Microsoft Excel^®^ spreadsheet and imported into STATA 10 (www.stata.com 2007) for data analysis. Cross-tabulation was performed in order to assess the association between variables. The level of statistical significance was set at < 0.05.^10^

### Ethical considerations

The Medunsa Research Ethics Committee of the University of Limpopo approved the study (Reference: MREC/H/99/2010: PG) and the permission to conduct the survey at the study site was obtained from the managers at the institution.

## Results

### Sociodemographic profile of participants

Of the 130 participants who received the questionnaire, 110 returned completed questionnaires – a response rate of 84.6%. The majority of participants were women (*n* = 66; 60%), married (*n* = 79; 71.8%) and nursing staff (*n* = 82; 74.5%). The mean age of participants was 30.76 ± 6.84 years old, with ages ranging from 20 to 56 years. Two age categories were created based on the median age of 30 years; the majority of participants (*n* = 64; 58.2%) were younger than 30 years of age, with 60 (54.5%) having five or less years of working experience, as is shown in Table 1.

**TABLE 1:** Sociodemographic details of the participants.

Variables	*n*	%
**Age category**		
< 30 years old	64	58.2
> 30 years old	46	41.8
**Gender**		
Male	44	40.0
Female	66	60.0
**Professional category**		
Medical doctors	12	10.9
Nurses	82	74.5
Pharmacists	9	8.2
Counsellors	7	6.4
**Work experience category**		
Five years or less	60	54.5
Over 5 years	50	45.5
**Marital status**		
Single	28	25.5
Married	79	71.8
Divorced	2	1.8
Widowed	1	0.9
**Knowledge level**		
Good knowledge level	52	47.3
Insufficient knowledge level	58	52.7

### Participants’ knowledge about multidrug-resistant tuberculosis

The mean knowledge score of the participants was 74% ± 14.3%, ranging from 40% to 100% of correct answers. Overall, less than half (47.3%) of participants had good knowledge about MDR-TB. Participants gave incorrect answers about what constituted MDR-TB, how it is diagnosed, and the duration of treatment. As shown in Table 2, the level of knowledge differed based on the age and professional category of the participants.

**TABLE 2:** Participants’ level of knowledge.

Variables	Good level of knowledge		Insufficient level of knowledge		Total	
	*n*	%	*n*	%	*n*	%
**Age category**						
Less than 30 years	29	45.3	35	54.7	64	100
30 years and above	23	50	23	50	46	100
**Gender**						
Male	21	47.7	23	52.3	44	100
Female	31	47	35	53	66	100
**Professional category**						
Doctors	10	83.3	2	16.7	12	100
Nurses	36	43.9	46	56.1	82	100
Pharmacists	4	44.4	5	55.6	9	100
Counsellors	2	28.6	5	71.4	7	100
**Work experience category**						
5 years or less	29	48.3	31	51.7	60	100
Over 5 years	23	46	27	54	50	100

There were no significant differences between male and female participants, or between those with different numbers of years of work experience (*p* > 0.05), as is shown in Table 3.

**TABLE 3:** Odd ratios of features associated with good knowledge level.

Variables	Odds ratio (95% CI)	*p*-value
Age (Over 30 years vs. < 30 years old)	1.21 (0.56, 2.59)	0.63
Working experience (< 5 years vs. > 5 years)	1.09 (0.51, 2.35)	0.81
Male versus female	1.03 (0.48, 2.23)	0.94
Doctors versus nurses	6.28 (1.42, 44.40)	0.01
Doctors versus pharmacists	5.66 (0.77, 57.56)	0.09
Doctors versus counsellors	10.46 (1.22, 132.40)	0.03

With regard to professional category, medical doctors showed a significantly better level of knowledge about MDR-TB than nurses and counsellors, but the difference was not significant when compared with pharmacists (*p* = 0.09).

### Participants’ practices with regard to multidrug-resistant tuberculosis

With regard to practice, about 83% (*n* = 91) of the participants stated that they used protective masks whilst attending to MDR-TB patients. About two-thirds (*n* = 73; 66.4%) of the participants reported being involved in educating patients about MDR-TB. Moreover, some 61.8% (*n* = 68) reported having their own copy of the MDR-TB treatment guidelines; and about 55% (*n* = 60) stated that they referred to these guidelines. These practices differed based on the professional category and other characteristics of the participants. For instance, 11 (91.7%) of the medical doctors and 74 (90.2%) of the nurses reported wearing protective masks; in contrast, only four (57.1%) of the counsellors and two (22.2%) of the pharmacists reported doing so. Moreover, pharmacists were the least involved in educating patients about MDR-TB – more than half of them (*n* = 5; 55.6%) reported not being involved. On the contrary, 71.4% (*n* = 5) of the counsellors were involved in educating patients as well as 69.5% (*n* = 57) of the nurses and 58.8% (*n* = 7) of the medical doctors.

It is noteworthy that participants with a good level of knowledge about MDR-TB were more involved in educating patients about the disease as compared with those with insufficient knowledge (*n* = 48, 75% versus *n* = 19, 58.5%; *p* = 0.07). In contrast, 41.2% (*n* =14) of participants with an insufficient level of knowledge about MRD-TB did not participate in educating patients, whilst a quarter of those with insufficient level of knowledge did not wear protective masks when attending to MDR-TB patients (*n* = 7, 25.9% versus *n* = 4, 7.7%; *p* = 0.01).

Furthermore, based on the professional category, all of the counsellors reported that they do not refer to the MDR-TB treatment guidelines, whereas three-quarters of the medical doctors (*n* = 9; 75%) reported doing so, as did 56.1% (*n* = 46) of the nurses and 55.6% (*n* = 5) of the pharmacists. Interestingly, it was found that participants with a good level of knowledge referred to the MDR-TB management guidelines more significantly than those with an insufficient level of knowledge (*n* = 50, 66% versus *n* = 15, 44.1%; *p* = 0.03). Overall, participants older than 30 years, men and those with more than five years of work experience, referred more to the MDR-TB treatment guidelines than their counterparts, but the differences were not of statistical significance (*p* > 0.05).

## Discussion

The findings from this study show that the majority of participants were women, young adults and nurses. This distribution is consistent with the composition of the healthcare workforce in Lesotho, where 73.3% are nurses and mostly women.^11^

In line with the purpose of the study, it is disappointing to note that less than half of the participants had a good level of knowledge about MDR-TB. The deficits in knowledge identified were about what constituted MDR-TB, how it is diagnosed and the duration of its treatment. These knowledge gaps are similar to what had been reported by other investigators.^4,5,8,12,13^

Based on personal characteristics such as age, gender and number of years of working experience, there is no evidence from this study to suggest that there was any statistically-significant difference amongst participants with regard to their knowledge of MDR-TB. However, based on professional category, more nurses and pharmacists had an insufficient level of knowledge as compared with medical doctors. This deficit in knowledge was even more pronounced amongst counsellors, as 71.4% of them had insufficient level of knowledge about MDR-TB. These findings are similar to reports by other investigators such as Kiefer et al.^5^ and Ahmed et al.,^12^ raising some concerns in that counsellors who educate patients about MDR-TB are themselves not so knowledgeable about it. This observation suggests that there is an urgent need for counsellors to be educated on MDR-TB and other aspects regarding tuberculosis treatment and care.

With regard to other practices relating to MDR-TB, given the usefulness of treatment management guidelines, it is unacceptable that about 40% of participants reported not having their own copy of the treatment guidelines. This situation is unacceptable because the guidelines are documents that every HCW should possess in order to ensure consistency and quality in the delivery of healthcare and services.^14,15,16^ It is important that institutional managers address this issue by making the guidelines available to all HCWs at the study site and at other healthcare facilities in the country.

With regard to the practice of using protective masks, although this study did not investigate whether these masks were purchased regularly and always available to staff members, about 17% of the participants reported that they did not used protective masks when they were in contact with MDR-TB patients. This is not acceptable, as all HCWs should use protective masks when dealing with MDR-TB patients. This is particularly important for pharmacists and counsellors who, routinely, are not provided with protective masks and are thus exposed to infection. This situation should be remedied because by protecting these HCWs, their family members and all other persons they interact with will be also protected.^17,18^

An important and encouraging finding from this study is that participants with good knowledge about MDR-TB were reported to use protective masks more significantly than those with an insufficient level of knowledge about MDR-TB (*p* = 0.01). This finding suggests that by increasing the level of knowledge it is possible to get a higher compliance with preventive interventions amongst HCWs. This is of utmost importance in order to avoid an increase in the burden of tuberculosis as an occupational disease amongst HCWs in Lesotho, as has been reported in other settings such as China.^19^

With regard to educating patients about MDR-TB, although two-thirds (66.4%) of the participants reported being involved in patient education, it is disconcerting that the majority of pharmacists were not involved in this important public heath activity. Whether this is due to increased workload or lack of initiative, it is critical that pharmacists participate in educating patients about MDR-TB, particularly regarding the pharmacotherapeutic aspects of the disease.^20^ This is even more important given the finding from this study that counselors, as community health workers with a high school education, displayed an insufficient level of knowledge about MDR-TB but were reportedly educating patients about the disease.

With regard to the practice of referring to the treatment guidelines, about 55% of the participants reported that they referred to them. This is a worrying finding; even more so in that none of the counsellors reported *ever* referring to these guidelines. Although this finding is consistent with a report by several other investigators who stated that a good number of healthcare practitioners fail to comply with clinical practice guidelines, this needs to be corrected.^21,22,23^ Although it is not established whether the guidelines manuals were purchased and made available to HCWs at the study site, institutional managers ought to ensure that clinical practice and management guidelines, including the MDR-TB treatment guidelines, should be made available to every HCW in the healthcare system. The above findings present the situation about MDR-TB at the time the study was conducted and may not be a permanent feature at the study site and throughout the country. Another limitation is that since this was a cross-sectional survey, causal relationships could not be established.

## Conclusion

In conclusion, the level of knowledge about MDR-TB amongst HCWs, especially amongst nursing and allied health staff members as compared to medical doctors at the study site, was not at an acceptable level. The insufficient level of knowledge was associated with unsafe practices, such as not wearing protective masks, as well as not owning and not referring to the MDR-TB treatment guidelines. An educational intervention is recommended as well as an allocation of funds for the purchase of required protective equipment and clinical practice management guidelines and manuals.
